# A Concurrent Validity Study of the Mullen Scales of Early Learning (MSEL) and the MacArthur-Bates Communicative Developmental Inventory (CDI) in Infants with an Elevated Likelihood or Diagnosis of Autism

**DOI:** 10.1007/s10803-024-06652-4

**Published:** 2025-01-17

**Authors:** Z. Belteki, E. K. Ward, J. Begum-Ali, C. van den Boomen, S. Bölte, J. Buitelaar, T. Charman, E. Demurie, T. Falck-Ytter, S. Hunnius, M. H. Johnson, E. J. H. Jones, I. Oosterling, G. Pasco, M. K. J. Pijl, A. Radkowska, M. Rudling, P. Tomalski, P. Warreyn, C. Junge, E. Haman

**Affiliations:** 1 Present Address: Faculty of Experimental Psychology, Helmholtz Institute, Utrecht University, Utrecht, Netherlands; 2 Department of Psychological Sciences, Birkbeck, University of London, London, England; 3 Department of Experimental Psychology, University College London, London, England; 4 Department of Psychological Sciences, Centre for Brain and Cognitive Development, Birkbeck, University of London, London, England; 5 Department of Psychology, Institute of Psychiatry, Psychology and Neuroscience, King’s College London, London, England; 6 Faculty of Psychology and Educational Sciences, Ghent University, Ghent, Belgium; 7 Donders Institute for Brain, Cognition and Behaviour, Radboud University, Nijmegen, Netherlands; 8 Karakter Child and Adolescent Psychiatry University Centre, Nijmegen, The Netherlands; 9 Department of Cognitive Neuroscience, Radboudumc, Nijmegen, The Netherlands; 10 Center of Neurodevelopmental Disorders (KIND), Department of Women’s and Children’s Health, Centre for Psychiatry Research, Karolinska Institute, Stockholm, Sweden; 11 Center for Psychiatry Research, Region Stockholm, Sweden; 12 Development and Neurodiversity Lab, Department of Psychology, Uppsala University, Uppsala, Sweden; 13 Faculty of Psychology, University of Warsaw, Warsaw, Poland; 14 Institute of Psychology, Polish Academy of Sciences, Warsaw, Poland; 15 Department of Psychology, University of Cambridge, Cambridge, England; 16 Child and Adolescent Psychiatry, Stockholm Health Care Services, Region Stockholm, Stockholm, Sweden; 17 Curtin Autism Research Group, Curtin School of Allied Health, Curtin University, Perth, Australia

**Keywords:** Language assessment, Communicative Developmental Inventory, Mullen Scales of Early Learning, Autism, Infants

## Abstract

Infants at elevated likelihood for or later diagnosed with autism typically have smaller vocabularies than their peers, as shown by the Mullen Scales of Early Learning (MSEL) and the MacArthur-Bates Communicative Developmental Inventory (CDI). However, the extent to which MSEL and CDI scores align remains unclear, especially across clinical and non-clinical populations. This study examined whether the concurrent validity of the MSEL and CDI differs based on autism likelihood and diagnosis. Data from 720 14-month-old infants were analysed, grouped by likelihood (elevated vs. typical) and diagnosis at 36 months (diagnosed vs. not diagnosed). Vocabulary scores were compared across both likelihood and diagnostic groups. Moderate correlations were observed between the MSEL and CDI in most groups (r_s_ range = [.34–.58]). One exception was that the expressive scores of elevated likelihood infants on the MSEL and CDI were more closely associated than the expressive scores of typical likelihood infants. Diagnosed infants had lower vocabulary scores than non-diagnosed peers on both the MSEL and CDI. The elevated likelihood group showed lower scores on the MSEL but not the CDI compared to typical likelihood infants. The moderate correlations suggest that the MSEL and CDI assess different aspects of language in infancy. These associations were weaker than previously reported in autistic children. Differences in vocabulary scores across likelihood and diagnostic groups highlight the need for further research to understand the association between these measures.

**Supplementary Information:**

The online version contains supplementary material available at 10.1007/s10803-024-06652-4.

Autism Spectrum Disorder is a developmental condition characterised by differences in social interactions and communication that are noticeable at an early age. Although language delay is no longer a criterion for diagnosis in the DSM-5, young children who later receive an autism diagnosis are frequently observed to have smaller expressive and receptive vocabularies than children who are not diagnosed with autism (Belteki et al., 2022; Boucher, 2003; Iverson et al., 2018). Infants who have a familial history of autism, meaning that they have an ‘elevated’ likelihood of diagnosis, are similarly shown to have delayed language development compared to typical likelihood peers (Charman et al., 2017; Marrus et al., 2018). It is important to investigate the group differences between autistic versus non-autistic infants, and between elevated versus typical likelihood infants, because the presence and quality of early language skills is an important predictor of the course of autism, and will also have an impact on interventions offered (e.g., speech and language therapy). Therefore, the accurate measurement of early language skills is important to determining language as well as later life outcomes (Bal et al., 2020; Swanson et al., 2019). This makes it vital to track the early language development of elevated likelihood and later diagnosed infants as a pointer to atypical developmental outcomes.

Language development during infancy can be assessed using a number of widely used measures, including the Mullen Scales of Early Learning (Bradley-Johnson, 1997; Mullen, 1995), a behavioural assessment completed by a researcher or clinician, and the MacArthur-Bates Communicative Developmental Inventory (CDI; Fenson et al., 2007), a parent or caregiver^1^ completed report. Despite the conceptual and methodological differences between the assessments, they are found to report similar group differences between elevated versus typical likelihood infants, and also between autism-diagnosed versus non-autistic infants (Belteki et al., 2022). However, there is reason to believe that the agreement between the measures could vary depending on an infant’s likelihood of diagnosis (elevated versus typical) and an infant’s later diagnosis (autistic versus non-autistic). This is because autism is a condition that can affect social interactions and communication from an early age, which can impact the accuracy of an assessment that aims to measure these very constructs (Jones et al., 2014; Landa et al., 2007).

The current paper aimed to examine whether the concurrent validity, or agreement, with which the MSEL and CDI assessed expressive and receptive vocabulary varied depending on the likelihood or diagnostic classification of the child. Infants were examined at 14 months of age, when they could comprehend the meaning of words but not yet express many of them, because receptive language typically precedes production (Frank et al., 2017, 2021; Kuhl, 2004). This age group was examined because prior to word production, it is more difficult to accurately assess the infant’s receptive language, as it is more challenging to define what constitutes understanding than it is to define what constitutes word production (Tomasello & Mervis, 1994). Measuring word understanding relies on attention to the behaviours of the child as a way to gauge their knowledge, and it may be even more challenging to interpret these behaviours when assessing elevated likelihood or later diagnosed infants, who may have atypical social and communication skills. Data for these analyses were taken from Eurosibs, a European consortium study (Jones et al., 2019), which followed infants with and without elevated likelihood of autism across multiple European sites.

The current earliest age for a diagnosis of autism spectrum disorder (ASD) is two years (Lord & Luyster, 2006), and this presents a challenge when studying the group characteristics of autistic infants during the first two years. Recently, collective efforts have been made to overcome this problem through cohort studies that build large datasets on infants with an elevated likelihood of autism diagnosis (Jones et al., 2014). Infants who are at an elevated likelihood of autism diagnosis have a family history of autism, meaning that a first-degree relative, such as an older sibling or parent, has been diagnosed with autism. This is found to increase an infant’s likelihood of autism diagnosis by roughly 20% (Ozonoff et al., 2011, 2024). By studying and comparing infants who are elevated versus typical likelihood, and then also retrospectively studying those who receive a diagnosis of autism versus those who do not, we can gain a clearer understanding of the markers in the first two years after birth that indicate a possibility of later autism diagnosis (Jones et al., 2014).

In the first two years after birth, there are observable differences between the two diagnostic groups and also between the two likelihood groups. Infants with a later diagnosis of autism show a decreased frequency of orientation to social stimuli, gesture use, and imitation when compared to their non-diagnosed peers (Baranek, 1999; Osterling et al., 2002). By 14 months, infants with a later diagnosis of autism are found to perform worse on a number of measures of social communication and play, including the frequency of gaze shifting between an object and a social partner’s eyes, the initiation of joint attention and gesture production (Landa et al., 2007). Similarly, elevated likelihood infants are shown to have impaired attentional disengagement and display more repetitive behaviours compared to their typical likelihood peers, despite being a more heterogeneous group that contains both later diagnosed and non-diagnosed infants (Canu et al., 2021; Iverson et al., 2018; Landa et al., 2007).

In the first few years of life, language abilities are frequently assessed using the Mullen Scales of Early Learning (MSEL) and the MacArthur-Bates Communicative Developmental Inventory (CDI) (Bradley-Johnson, 1997; Fenson et al., 2007). Both measures are standardised and demonstrate good validity and reliability in assessing the language abilities of infants in the first years of life (Riley et al., 2019; Thal et al., 2007). The MSEL is a researcher- or clinician-completed behavioural assessment that is carried out in a laboratory environment. The MSEL assesses expressive and receptive abilities more broadly than the CDI, going beyond the word knowledge by assessing behaviours such as voluntary babbling (Bradley-Johnson, 1997). The CDI is a parent-completed report that is filled out in the home environment. It zooms in on infants’ expressive and receptive abilities using an extensive checklist where the specific words that the infant understands and/or produces can be ticked off (Fenson et al., 2007).

Despite their differences, studies conducted with either the CDI or MSEL on elevated likelihood and later diagnosed groups of children concur that these children have on average lower language scores compared to their typical likelihood and non-diagnosed peers (Belteki et al., 2022; Kwok et al., 2015). In addition, both measures also have good concurrent validity with other assessments of language and social development, such as the Differential Ability Scales for nonverbal and verbal IQ scores and the Language Development Survey (Farmer et al., 2016; Rescorla et al., 2005). To date, one study has directly assessed the concurrent validity between the MSEL and CDI (Nordahl-Hansen et al., 2014). This study found high agreement between the two measures in 2- to 4-year-old autistic infants, with a Spearman’s rank order ranging from 0.81 to 0.95 for expressive and receptive language measures. This high concurrent validity indicated that the language score collected on one measure could be indicative of the language scores collected on the other measure, despite their methodological and conceptual differences.

Although there is some evidence of a high concurrent validity between the MSEL and CDI (Nordahl-Hansen et al., 2014), the challenges arising from assessing language levels in autistic infants create a need to further assess the validity of these language measures (Akshoomoff, 2006; Charman et al., 2003). If there are group-related differences in the concurrent validity of the MSEL and CDI, this could suggest that for certain infants, the scores of one assessment may not be indicative of the scores they would receive on another assessment that similarly assesses language, but differs conceptually and methodologically.

Elevated likelihood and later diagnosed infants both exhibit atypical behaviours in the first years after birth, and these group-level differences in the first years of life may influence the accuracy with which their language abilities can be assessed. It is important to investigate the accuracy of language assessments, because early language abilities can influence the interventions that are offered to children (e.g., speech and language therapy), along with their later life outcomes (Bal et al., 2020; Swanson et al., 2019). In a previous study by Akshoomoff (2006), it was found that the behavioural difficulties in autistic infants had an impact on direct behavioural assessments like the MSEL. The study coded the overt behaviours of 16- to 43-month-old autistic infants and their typically developing peers, during MSEL observations. It was observed that autistic infants spent significantly less time engaging with the assessment than the typically developing group. In turn, the time spent engaging with the task was found to relate to lower scores on the MSEL. The level of engagement may be particularly important in a behavioural assessment such as the MSEL, where the researcher or clinician completing the assessment has limited time in which to assess a child they have little to no prior experience with. Consequently, the characteristics of the child, such as a shorter attention span or a shy response to strangers, could reduce the reliability of such behavioural assessments (Aldridge et al., 2017; Chiat & Roy, 2007; DesChamps et al., 2020; Feldman et al., 2000).

Similar concerns about behaviours affecting the accuracy of the assessment have been raised for the CDI (Charman et al., 2003). Certain aspects of development, such as receptive vocabulary, are thought to be more difficult to assess in autistic infants because of their lower inclination to orient to social cues (Charman, 2004). Autistic children’s reduced inclination to engage has been observed during parent–child interactions in the home environment, with a study from Del Rosario et al. (2023) finding that 6-month-old elevated likelihood infants had a lower engagement intensity with their parents than typical likelihood peers (Del Rosario et al., 2023). These differences in the length and the quality of the engagement may reduce the accuracy with which elevated and later diagnosed infants can be assessed compared to their typical likelihood and non-diagnosed peers. This may particularly impact an assessment such as the CDI, where parents assess their infants in a less standardised way. Although all parents receive the same instructions, these are open for different interpretations. For example, parents can tick a word as ‘understood’ and/or ‘said’ when instead of the word a synonym is used or when the child attempts to produce the word, respectively. This can lead to the parents’ beliefs having more impact on the outcomes of the questionnaire (Feldman et al., 2000; Hart & Risley, 1995). In turn, if an infant’s performance on the assessments is over- or underestimated by their parents, it may lower the accuracy of CDI assessments and impact how high the concurrent validity between the MSEL and CDI is. However, whether the concurrent validity across the measures differs across likelihood and diagnostic classification groups and whether other factors (such as the language of testing) impact cross-measure association is yet to be explored.

The current study aimed to examine the concurrent validity between MSEL and CDI scores of infants who were elevated likelihood, typical likelihood, later autism diagnosed, and later non-diagnosed. We focused on data from these groups at 14 months of age, when the final diagnostic classification of the infants as autistic or non-autistic was not yet determined. We hypothesised that agreement between the measures would be lower for elevated likelihood and later autistic diagnosed infants, due to group differences in the social and communicative abilities of elevated versus typical likelihood, and autistic versus non-autistic infants, which may influence the accuracy of the assessment (Charman, 2004). We also analysed whether the group effects on the concurrent validity between the MSEL and CDI differed depending on the country in which the testing was carried out. We hypothesised there would be consistent patterns across countries, as both the MSEL and CDI have been adapted for all the countries from which we collected data. Assessing whether infant characteristics have an impact on the association between parent reports and behavioural assessments can provide us with a clearer understanding of how accurately we can assess a child’s language ability with the current gold-standard measures, and whether this accuracy is contingent on the group classification of the child.

## Methods

The data were obtained from the Eurosibs cohort (Jones et al., 2019), which was a European consortium on elevated likelihood/autistic and typical likelihood/non-autistic infant populations, using a longitudinal cohort research design. Infants were classified as ‘elevated likelihood’ if their older sibling had a diagnosis of autism,^2^ because having a first degree relative with a diagnosis of autism elevates the likelihood that a child will receive a diagnosis themselves by roughly 20 times (Ozonoff et al., 2011, 2024). To be classified as ‘typical likelihood’, infants needed to have an older sibling who was typically developing with no known genetic or developmental disorders, and no first-degree relatives with an autism diagnosis. Post-diagnostic classifications were made based on the DSM-5 best estimate research diagnosis that the infants received at 36 months, by using both Autism Diagnostic Observation Schedule (ADOS-2; Lord et al., 2000), Autism Diagnostic Interview-Revised (ADI-R; Rutter et al., 2003), MSEL (Mullen, 1995) and the Vineland (Sparrow et al., 2005). Data was collected from four countries: the Netherlands (Radboud University in Nijmegen and Utrecht University in Utrecht); Poland (University of Warsaw in Warsaw); Sweden (Uppsala University in Uppsala), and the United Kingdom (King’s College London and Birkbeck, University of London, both in London). All sites followed the same process. For both the likelihood and diagnostic groups, we aimed to use the most up-to-date and preferred terminology for the groups, based on recent studies (Keating et al., 2023).

## Participants

In the likelihood groups, data were collected from 720 14-month-olds (225 typical likelihood). Of these, 82.78% (n = 596) of infants returned for final diagnosis at the post-diagnostic stage (496 non-autistic). In line with existing findings, roughly 20% of the elevated likelihood group went on to receive a diagnosis of autism at 36 months of age (Ozonoff et al., 2011, 2024). Infants were grouped based on likelihood as well as diagnosis, because elevated likelihood infants are also shown to differ in their social and communicative abilities from typical likelihood peers (Charman et al., 2017; Landa et al., 2007; Marrus et al., 2018). A breakdown of the age of the infants per site can be found in Table 1. Protocols were approved by the relevant ethics committee at each site and were conducted in accordance with the Declaration of Helsinki and the American Psychological Association (Jones et al., 2019). Parents provided informed consent. Descriptive information about the dataset including a child’s sex, language experience, and parental education is provided in Table 2.

**Table 1:** The age groups of the infants, per site, in months

	Likelihood groups		Diagnostic groups	
Site of testing	Typical likelihood	Elevated likelihood	Non-autistic	Autistic
Netherlands (n = 115)	*M *= 14.36, *SD* = 0.56	*M *= 14.19, *SD* = 0.49	*M *= 14.24, *SD* = 0.53	*M *= 14.23, *SD* = 0.41
Poland (n = 54)	*M *= 14.84, *SD* = 0.69	*M *= 14.66, *SD* = 0.85	*M *= 14.75, *SD* = 0.80	*M *= 14.34, *SD* = 0.80
Sweden (n = 174)	*M *= 14.21, *SD* = 0.64	*M *= 14.22, *SD* = 0.57	*M *= 14.17, *SD* = 0.59	*M *= 14.41, *SD* = 0.54
United Kingdom (n = 377)	*M *= 14.34, *SD* = 1.30	*M *= 14.66, *SD* = 1.36	*M *= 14.49, *SD* = 1.39	*M *= 14.61, *SD* = 1.37

**Table 2:** The characteristics of the likelihood and diagnostic groups

Characteristics	Typical likelihood (n = 225)	Elevated likelihood (n= 495)	Non-autistic (n= 496)	Autistic (n = 100)
Sex, %				
Male	48.89%	51.71%	47.98%	65.00%
Female	50.67%	48.08%	51.81%	35.00%
Not reported	0.40%	0.20%	0.20%	0.00%
Child language experience, % (*)				
Monolingual	66.22%	58.00%	59.48%	54.00%
Multilingual	10.67%	12.32%	10.69%	11.00%
Not reported	23.11%	29.70%	24.19%	35.00%
Parental education, % (^)				
Primary	0.00%	0.81%	0.40%	2.00%
Secondary	13.33%	29.90%	21.37%	38.00%
Tertiary	70.22%	49.70%	61.90%	32.00%
Not reported	16.44%	19.60%	16.33%	28.00%

The characteristics of the likelihood and diagnostic groups

NB (*): Infants were defined as multilingual if they heard a language in their home at least 20% of the time that was not the majority language of the relevant country (Deanda et al., 2016)

(^): This is the highest level of education completed by either of the parent who provided the majority of care. In the datasets where a main caregiver was not defined, the highest education level of the mother was used.

## Materials and Procedure

### Mullen Scales of Early Learning (MSEL)

The Mullen Scales of Early Learning (or MSEL) can be administered with infants from birth to 68 months of age (Bradley-Johnson, 1997), and it is made up of five subtests: visual reception, fine motor, gross motor, expressive language, and receptive language (Mullen, 1995). The measure shows high construct validity as well as high concurrent validity with other measures of language ability, such as the Bayley Scales of Infant Development (Bayley, 2006; Mullen, 1995). In this paper, we focused on the ‘expressive language’ and ‘receptive language’ subscales of the assessment. An example of an item on the expressive language scale is checking for infants’ voluntary babbling. An example of an item on the receptive language scale is checking that infants attends to words and movement. Every site used the US version of the MSEL, which was translated by the testers to the local language. This was done because there is no language measure available that is cross-standardised in the EU countries involved in the study. To ensure interrater reliability, cross-site consensus meetings were regularly organised, in which MSEL assessments were coded and discussed together. The MSEL was administered by trained researchers and followed strict guidelines. All testers completed training, which included role-playing, scoring videos, and achieving 90% reliability across three supervised assessments. These assessments were validated by an experienced clinician or researcher. We also carried out additional checks in this paper to examine whether different results were observed for English participants versus non-English participants, who were tested on a translated version of the MSEL (see Supplementary material D).

During the testing phase, the testers were aware of the infant’s likelihood classification. For the analyses, basic performance scores (or raw scores) were tallied separately for each of the subscales.

### The MacArthur-Bates Communicative Developmental Inventory (CDI)

At 14 months, infants were tested on the ‘Words and Gestures’ version of the CDI (CDI-WG). This is a list of 380–652 words (depending on the language version), which are organised into semantic categories, such as ‘Games and Routines’ or ‘Toys’. From the vocabulary checklist in this CDI, we calculated expressive vocabulary by tallying the number of words that infants ‘Understand and speak’, and we calculated receptive vocabulary by tallying the number of words that infants ‘Understand only’ and ‘Understand and speak’. Each site used the appropriate language version of the CDI: British English, Dutch, Polish, and Swedish. All different versions of the CDI-WG used in this paper have been validated in the languages that they are used; British English (Alcock et al., 2020); Dutch (Zink & Lejaegere, 2002); Polish (Smoczyńska et al., 2015); and Swedish (Eriksson & Berglund, 1999).

## Analyses

All statistical analyses were run in R, version number 4.3.0 GUI 1.79 (R core team, 2023), RStudio, version 1.4.1103 (R Studio team, 2023) and SPSS, version 29.0.0.0 (IBM Corp., 2023). We started by assessing whether there were significant differences in expressive and receptive scores of the infant groups that were comparable to the existing findings in the literature (Belteki et al., 2022; Kwok et al., 2015). The Mann–Whitney U test was used as a non-parametric alternative to the parametric independent sample t-test, because the data violated some of the assumptions required for parametric testing. Comparisons were made between groups for both MSEL raw scores and CDI raw scores.

To assess the association between the MSEL and CDI per group, we compared the correlations between the raw scores of the measures, for both receptive ability and expressive ability. For this we used Spearman’s Rho, a non-parametric equivalent to Pearson’s correlation, as the data was often skewed. Commonly, the Spearman’s Rho correlations are interpreted as follows: an index *r*_*s*_ (or correlation) between 0.40–0.59 indicates moderate association, 0.60–0.79 indicates high association, and 0.80–1.0 indicates very high association (Nordahl-Hansen et al., 2014). Next, we contrasted the Spearman’s Rho correlations between groups using Fisher’s Z transformations, both in the likelihood groups (typical versus elevated likelihood) and in the diagnosed groups (non-autistic versus autistic). We also examined the correlations separately for each country from which data was collected.

The group comparisons described above were made using the raw scores of the infants on the MSEL and CDI. However, across countries the length of the CDI varied. We therefore ran additional analyses where we eliminated these differences in scale by converting the CDI scores to proportions (see Supplementary materials A). We also checked whether the removal of scores below the 1st percentile or above the 99th percentile led to different outcomes from the analyses in the main body of the paper (see Supplementary materials B). We also ran additional analyses to examine whether gender influenced the group-related differences in concurrent validity. The results can be found in Supplementary materials C.

## Results

### Group Differences in Language Scores

We first assessed whether the group differences in expressive and receptive scores were comparable to the existing literature (Belteki et al., 2022; Kwok et al., 2015). Consequently, we compared expressive and receptive scores between the elevated versus typical likelihood groups and then the autistic versus non-autistic diagnostic groups. We compared these scores using the non-parametric Mann–Whitney U test and included the effect sizes for each analysis, which were as follows: 0.01—small effect size; 0.06—medium effect size, 0.14—large effect size (Cohen, 1988).

First, we compared the likelihood groups (elevated likelihood children versus typical likelihood children). For the CDI, the scores of typical likelihood infants were not significantly higher than the scores of elevated likelihood infants, neither for expressive language (*z* = − 0.127, *p* = 0.90, η^2^ = 0.0051), nor for receptive language (*z* = − 1.52, *p* = 0.129, η^2^ = 0.061).

For the MSEL, the scores of typical likelihood infants were significantly higher than those infants with an elevated likelihood of autism, both for expressive language (*z* = − 3.017, *p* = 0.003, η^2^ = 0.12), and for receptive language (*z* = − 2.72, *p* = 0.007, η^2^ = 0.11).

Next, we contrasted our diagnostic groups (autistic versus non-autistic children). For the CDI, the scores of non-autistic infants were significantly higher than those of autistic infants, both for expressive language (*z* = − 3.52, *p* < 0.001, η^2^ = 0.15), and for receptive language (*z* = − 3.67, *p* < 0.001, η^2^ = 0.16).

For the MSEL, the scores of non-autistic infants were significantly higher than the scores of autistic infants both for expressive language (*z* = − 5.31, *p* < 0.001, η^2^ = 0.23), and for receptive language (*z* = − 3.98, *p* < 0.001, η^2^ = 0.17).

## Correlations Between the MSEL and CDI Assessments

**Fig. 1:**
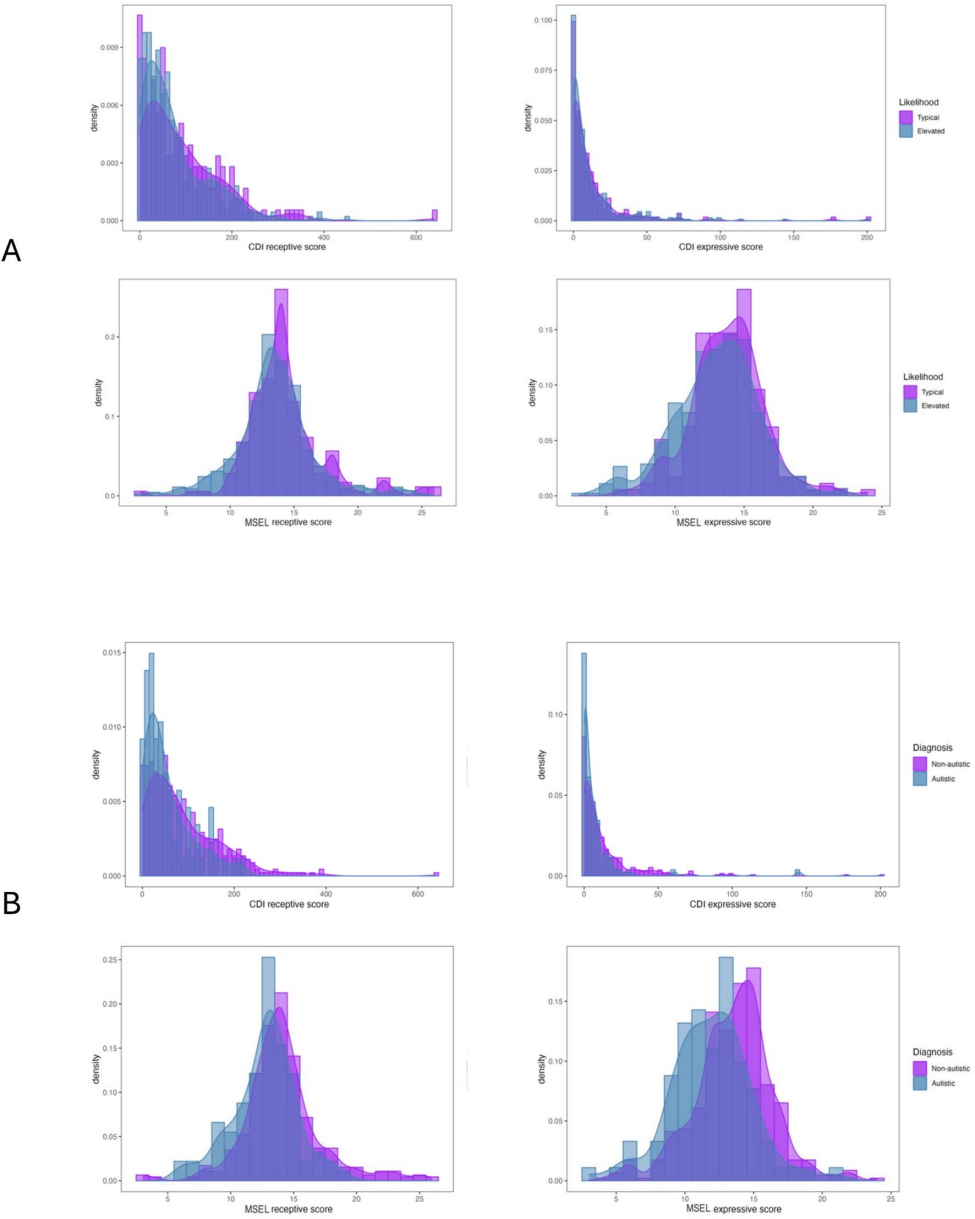
Histograms showing the density of the scores for the Mullen Scales of Early Learning (MSEL) and the MacArthur-Bates Communicative Developmental Inventory (CDI) in the likelihood (elevated or typical) groups (1**A**) and in the diagnostic (autistic or non-autistic) groups (1**B**), respectively

Figure 1 plots the distributions for both the likelihood groups (1A) and the diagnostic groups (1B) for each of our 4 dependent measures: the CDI expressive score; CDI receptive score; MSEL expressive score, and MSEL receptive score. While the CDI scores show more floor effects, especially in expressive language abilities, the MSEL scores appear more symmetrical for both expressive and receptive language. Additionally, while the CDI data has a positive skew, in particular for expressive language, the MSEL data appears to be largely normally distributed.

**Fig. 2:**
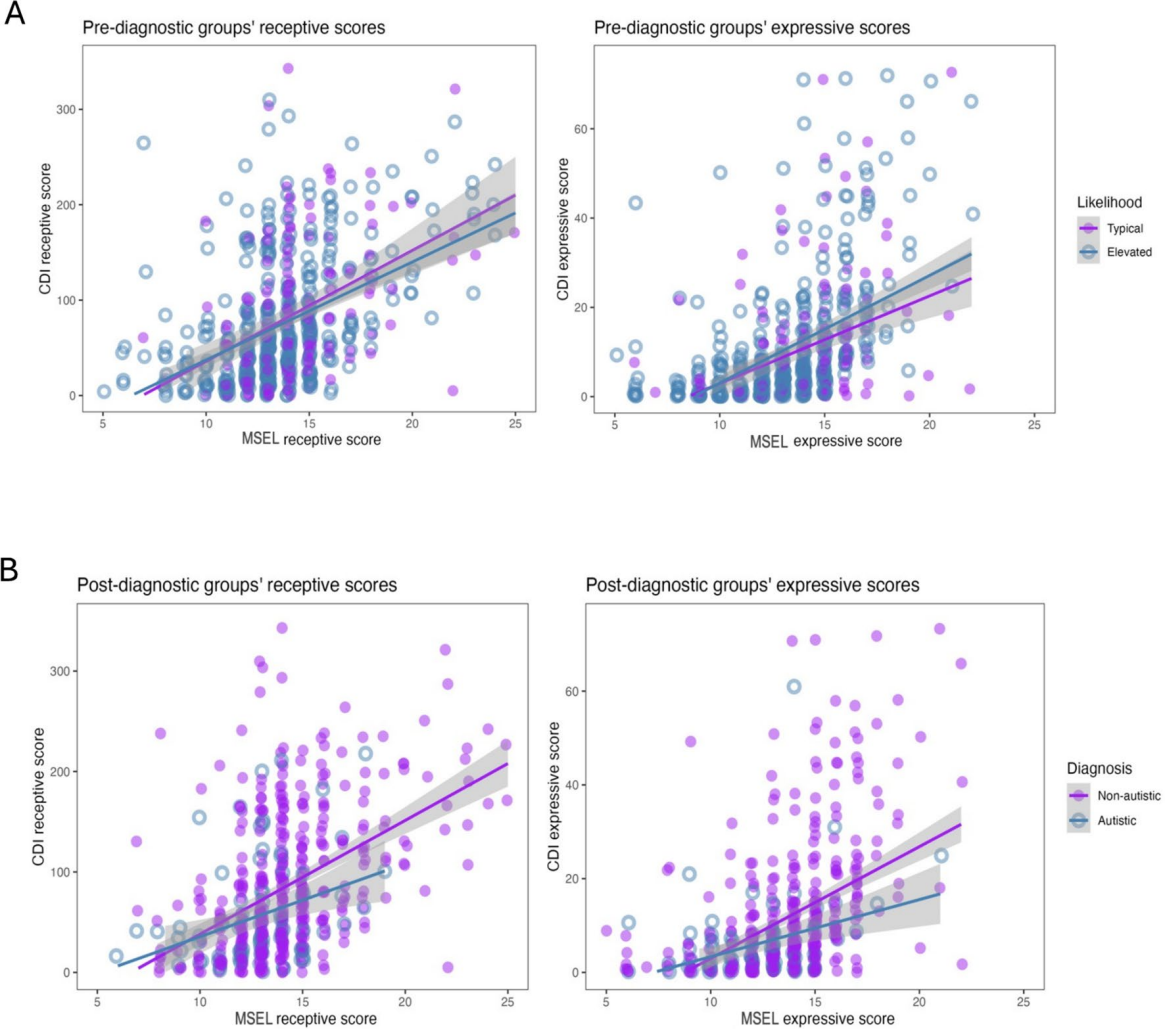
The correlations between the for the Mullen Scales of Early Learning (MSEL) and the MacArthur-Bates Communicative Developmental Inventory (CDI) for expressive scores and receptive scores in the likelihood (elevated or typical) groups (2**A**) and in the diagnostic (autistic or non-autistic) groups (2**B**), respectively

We calculated the correlations between the measurement of the MSEL and CDI separately for both expressive and receptive language, for both of the likelihood groups and for both diagnostic groups, using Spearman’s Rho _(_*r*_*s*)_. We then compared correlations between the typical compared to elevated likelihood group, and the non-autistic compared to autistic group, using the Fisher’s Z test. For illustrations of the correlations between measures, see Fig. 2A (the likelihood groups) and Fig. 2B (the diagnostic groups). Finally, we looked at whether the group differences in the association between the MSEL and CDI differed depending on the country (or site) in which the testing was carried out.

### Likelihood Group Comparisons

When examining the expressive scores of the typical likelihood group, there was a moderate positive correlation between the MSEL and CDI, *r*_*s*_(170) = 0.45, *p* < 0.001. For the elevated likelihood group, there was also a moderate positive correlation between the MSEL and CDI, *r*_*s*_(422) = 0.58, *p* < 0.001. The Fisher’s Z test showed that the correlation for the elevated likelihood group was significantly higher than that for the typical likelihood group, *z* = − 1.9, *p* = 0.029.

When examining the receptive scores of the typical likelihood group, there was a moderate positive correlation between the MSEL and CDI, r_*s*_(170) = 0.42, *p* < 0.001. For the elevated likelihood group, there was also a moderate correlation between the MSEL and CDI, r_*s*_(423) = 0.46, *p* < 0.001. The Fisher’s Z test showed that the correlations were not significantly different between the typical likelihood and elevated likelihood groups, *z* = − 0.66, *p* = 0.26.

### Diagnostic Group Comparisons

When examining the expressive scores of the non-autistic group, there was a moderate positive correlation between the MSEL and CDI, *r*_*s*_(433) = 0.55, *p* < 0.001. For the autistic group, there was also a moderate correlation between the measures, *r*_*s*_(83) = 0.44,* p* < 0.001. The Fisher’s Z test showed that the correlations were not significantly different between the non-autistic and autistic groups, *z* = 1.15, p = 0.13.

When examining the receptive scores of the non-autistic group, there was a moderate positive correlation between the MSEL and CDI, *r*_*s*_(434) = 0.46, *p* < 0.001. For the autistic group, there was a low correlation between the MSEL and CDI, *r*_*s*_(83) = 0.34, *p* = 0.002. The Fisher’s Z test showed that the correlations did not differ significantly between the non-autistic and autistic groups, *z* = 1.21, *p* = 0.11.

## Cross-Site Differences in Correlations

Information about the correlations per site are provided in Tables 3 (expressive scores) and 4 (receptive scores). Results show that for most sites, there was a moderate correlation between the MSEL and CDI scores in both the likelihood groups and in the diagnostic groups. For the Polish site, a significant difference was observed in the Spearman’s Rho (*r*_*s*_) between the two likelihood groups: the elevated likelihood group had higher association between their MSEL and CDI measures (*r*_*s*_ = 0.56) than the typical likelihood group (rs = − 0.13).

**Table 3:** The association between the MSEL and CDI for expressive scores, split by site

Expressive vocabulary	Likelihood groups			Diagnostic groups		
Site	Typical likelihood	Elevated likelihood	Group difference	Non-autistic	Autistic	Group difference
Netherlands	*rs*(23) = .58*	*rs* (41) = .57**	*z* = .06	*rs* (47) = .73**	N/ A^#^	N/A
Poland	*rs* (10) = .38	*rs* (17) = .56*	*z* = − .53	*rs* (26) = .40*	N/A^#^	N/A
Sweden	*rs* (38) = .51**	*rs* (111) = .52**	*z* = − .07	*rs* (121) = .55**	*rs* (28) = .36	*z* = 1.12
UK	*rs* (95) = .40**	*rs* (249) = .61**	*z* = − 2.34*	*rs* (235) = .53**	*rs* (43) = .49**	*z* = .33

NB: *marks significance at p < .05) and **at p < .01

^#^Analysis was not run because the number of included participants was < 10

**Table 4:** The association between the MSEL and CDI for receptive scores, split by site

Receptive vocabulary	Likelihood groups			Diagnostic groups		
Site	Typical likelihood	Elevated likelihood	Group difference	Non-autistic	Autistic	Group difference
Netherlands	*rs*(23) = .16	*rs* (41) = .39*	*z* = − .92	*rs* (47) = .43*	N/A ^#^	N/A
Poland	*rs* (10) = − .13	*rs* (17) = .56*	*z* = − 1.73*	*rs* (26) = .37	N/A^#^	N/A
Sweden	*rs* (38) = .52**	*rs* (111) = .55**	*z* = − .22	*rs* (121) = .54**	*rs* (28) = .48*	*z* = − .37
UK	*rs* (95) = .37**	*rs* (250) = .40**	*z* = − .29	*rs* (236) = .37**	*rs* (43) = .25	*z* = − .79

NB: ^*^marks significance at p < .05) and **at p < .01

^#^Analysis was not run because the number of included participants was < 10

## Discussion

The aim of this study was to assess whether the concurrent validity between the MSEL and CDI, two widely used measures of expressive and receptive language in infancy, varied depending on the likelihood for and later diagnosis of autism. We first carried out group comparisons to assess whether the difference in our sample of elevated versus typical likelihood infants, and later diagnosed versus non-diagnosed infants were comparable to what has been reported in the literature (Belteki et al., 2022; Garrido et al., 2017). In line with the existing literature, the group differences between the autistic and non-autistic infants were larger than the group differences between the elevated likelihood and the typical likelihood infants. We then looked at the concurrent validity between the MSEL and CDI in both likelihood groups and both diagnostic groups. Generally, moderate associations were found between the MSEL and CDI. This was also found when running the analyses separately for each country of testing.

## Mean Group Differences

When comparing the two likelihood groups, we found that the elevated likelihood infants did have a significantly lower expressive and receptive scores compared to typical likelihood infants when the MSEL was used as an assessment, but not when the CDI was used. In the diagnostic groups, autistic infants had significantly lower expressive and receptive scores compared to non-autistic infants both when the MSEL and when the CDI were used. Additionally, we observed larger group differences (as reflected in effect sizes) between the diagnostic groups than between the likelihood groups, which is in line with results of a recent meta-analysis (Belteki et al., 2022). These findings were similarly observed when the CDI scores were converted to proportions (see Supplementary materials A) and when the outliers from both the MSEL and CDI were removed (see Supplementary materials B). Overall, our findings are in line with the existing literature, which has frequently found that typical likelihood/non-diagnosed infants score higher than elevated likelihood/diagnosed infants on expressive and receptive measures (Belteki et al., 2022; Garrido et al., 2017).

A recent meta-analysis by Belteki et al. (2022) found a similar magnitude of difference between likelihood groups for both MSEL and CDI scores. This current study however only observed differences in the MSEL scores of typical and elevated likelihood infants, and not in their CDI scores. In contrast, differences were observed between the autistic and non-autistic infants on both the MSEL and the CDI. These differences in the results of the likelihood groups compared to the diagnosed groups were not linked to the raters’ knowledge of the diagnostic outcomes of the infants, because the diagnostic group classifications are carried out after 14 months, at 36 months. Instead, it is possible that the heterogeneity of the elevated likelihood sample (where the majority of children do not receive a diagnosis of autism later; Ozonoff et al., 2011, 2024) was too large. This large heterogeneity meant it was not possible to capture consistent patterns of language delays in the elevated likelihood group, meaning that the magnitude of difference was smaller between the elevated and typical likelihood group. It is also possible that differences between the elevated and typical likelihood groups in their vocabulary scores (as measured by the CDI) are not yet observable at 14 months of age. The CDI measures vocabulary outcomes, which is known to be highly variable at 14 months of age, even across individuals in the same group (Frank et al., 2017). Group differences in vocabulary outcomes may take longer to emerge than differences in language-related behaviours (as measured by the MSEL) which show less individual variation within groups. Furthermore, the vocabulary outcomes of elevated and typical likelihood infants overlaps substantially, because a large portion of the elevated likelihood group does not receive a diagnosis of autism at 36 months.

## Moderate Associations Across Infant Measures

Overall, the concurrent validity between the MSEL and CDI was moderate for all likelihood and diagnostic groups. This was also observed when the CDI was converted to proportions to account for cross-site differences in the length of the CDI (see Supplementary Material A), and when the outliers from both the MSEL and CDI were removed to determine the extent to which very low or high scores affected the results (see Supplementary Material B). Additionally, similar patterns of results were observed when the data was split based on gender, language spoken, and socio-economic status (see Supplementary materials C, D, and E).

The moderate associations for 14-month-olds in our study differ from a previous study looking at the concurrent validity of the CDI and MSEL in 24-month-olds, which observed very high agreement between the measures (Nordahl-Hansen et al., 2014). In part, the lower agreement in this paper may have resulted from the younger age group which was assessed. Previous studies have noted the challenges of accurately assessing younger infants, whose attention span and responsiveness tends to be lower (Aldridge et al., 2017; Chiat & Roy, 2007; DesChamps et al., 2020; Feldman et al., 2000).

Another challenge of assessing younger infants is that their vocabularies contain a greater proportion of words that are understood, but not yet produced. Assessing receptive language is difficult because it requires more interpretation than assessments of spoken language. This can lead to lower accuracy of assessment (Tomasello & Mervis, 1994). Similarly, it may be more challenging to assess expressive vocabulary in younger infants, who produce less speech. When infants say fewer words, parents may use techniques like elicited imitation to gauge their vocabulary. In this method, an adult says a word and checks if the child repeats it. While parents might consider imitation as a sign of expressive vocabulary, it lacks the communicative intent found in spontaneous speech. Consequently, infants might repeat words without fully understanding them, leading to inaccurate assessments of their expressive vocabulary (McDade et al., 1982; Vinther, 2002).

Eliciting words from infants and making interpretations of their expressive and receptive vocabulary may be more challenging for parents completing the CDI than for researchers completing the MSEL, because parents receive less standardised instructions on how to assess the words their infants understand and produce. However, the accuracy of behavioural assessments may also be lower when assessing younger infants, whose behaviours are more context-dependent. Younger infants have shorter attention spans, and their performance on assessments varies more across situations (Akshoomoff, 2006; Charman, 2004; Colombo, 2001; Rose et al., 1975). The MSEL may therefore have lower accuracy when assessing younger infants because it is completed in controlled settings, by a researcher or clinician without prior experience of the child.

Another explanation for the lower association between the MSEL and CDI compared to Nordahl-Hansen et al. (2014) is that the measures at this age differ in the components of expressive and receptive language that they assess, and that these components relate less to each other in infants than in toddlers. The CDI focuses solely on vocabulary: the typical early words and phrases that infants may understand or produce. In contrast, the items on the MSEL focus more on observable behaviours during interactions with others that indicate a capacity for expressive and receptive vocabulary, such as attention to the words and movements of parents. Association between the measures in older infants may be higher because, at a later stage of development, the behavioural skills leading to vocabulary growth (MSEL) may be more related to the number of words infants understand and produce (CDI). Yet for infants as young as 14 months, who are starting to build a vocabulary, these processes may not yet be entwined. More research is required to confirm this observation and understand its implications. Future research could, for instance, compare these two measures against other measures that capture either early communication behaviours more generally or focus on words exclusively (for example the Ages and Stages Questionnaire (Bricker et al., 1999); Bayley Scales of Infant and Toddler Development (Bayley, 2006); and the Reynell Developmental Scales (Edwards et al., 2011).

A notable observation of this study is that across all infant groups moderate associations were observed between the MSEL and CDI. These were present for infants with an elevated or typical likelihood of autism, and infants with a later diagnosis or no diagnosis of autism, across all sites, for both expressive and receptive vocabularies. These similarities warrant further discussion.

### Moderate Associations Across All Infant Groups

The moderate correlations across the majority of the infant groups suggests that the association between the MSEL and CDI is not impacted by the group classification of the infant. The differences in the social and communicative characteristics of the groups do not appear to have a significant effect on how reliably expressive and receptive vocabularies are assessed on the MSEL and CDI. Thus, this study does not find evidence that the assessment of elevated likelihood and autism diagnostic infants is less accurate than the assessment of typical likelihood and non-diagnosed infants.

However, there was one difference in the association observed between the groups—when assessing expressive vocabulary. The association between the MSEL and CDI was significantly higher for the elevated likelihood group than for the typical likelihood group. We had hypothesised an effect in the opposite direction—that typical likelihood infants would have a higher association between the MSEL and CDI. Although infants with elevated likelihood are at increased probability of a later autism diagnosis, the majority of this group (roughly 80%) will not develop autism, making this a very heterogeneous group in language skills. We are cautious in interpreting this group-related finding as meaningful, because this finding was the only significant deviance between the typical and atypical groups, and further the cross measure association was of a similar size—positive but moderate—in all of the likelihood and diagnostic groups.

### Moderate Associations for Both Expressive and Receptive Language

When comparing the group differences in vocabulary size, we observed in the CDI that the infants had smaller expressive than receptive vocabularies. This was an expected finding for this age group, as 14-month-olds have previously been shown to understand more words than they are yet able to produce (Bates et al., 1994; Braginsky et al., 2019; Fenson et al., 1994; Frank et al., 2017). In contrast however, on the MSEL the expressive and receptive scores of the infants were comparable to each other. We initially predicted that associations might be higher for expressive vocabularies, as expressive behaviours are easier to capture (Tomasello & Mervis, 1994), resulting in higher validity. Yet, both for receptive and for expressive vocabularies, the associations were comparable and moderate. Both expressive and receptive vocabulary may have been moderate, but for different reasons. Associations may have been moderate for expressive vocabulary due to the large prevalence of floor effects in expressive vocabulary in 14-month-olds for the CDI: this suggests that there is simply not enough variation between infants, while there is enough variation in the MSEL scores to capture some differences. In contrast, associations for receptive vocabulary measures may have been moderate due to the lower construct validity of the CDI (i.e., whether children truly comprehend the words they are indicated to; Houston-Price et al., 2007; Tomasello & Mervis, 1994). Future research could investigate whether the associations between the MSEL and CDI for expressive and receptive vocabulary remains consistent in older infants, or whether expressive vocabulary shows higher association once infants become older and produce more words. For 14-month-olds, the results suggest that association between the MSEL and CDI are comparable across expressive and receptive vocabulary.

### Moderate Associations Across European Research Sites

The associations between the CDI and the MSEL were found to be mostly comparable when they were examined per site, ranging from low to moderate associations. Crucially, group differences, both between likelihood groups and between diagnostic groups, were generally absent across the sites. There were however two exceptions to this: in the Polish dataset, when receptive vocabulary was measured, the association between the MSEL and CDI was significantly lower in the typical likelihood group than in the elevated likelihood group. However, this finding may be attributed to the small sample size that was available for this site, as in smaller samples, the magnitude of a correlation can be unstable (Schönbrodt & Perugini, 2013). The second exception was in the UK dataset: when expressive vocabulary was measured, the association between the MSEL and CDI was significantly higher in the elevated likelihood group than in the typical likelihood group. This result was also observable in Supplementary material D, where the difference between elevated and typical likelihood infants’ concurrent validity scores was only observable in the English speaking group. In part, these findings may be explained by the high variability of the elevated likelihood group (see previous point), which leads to higher extremes in the scores, on either distribution tail of both the MSEL and CDI, leading to higher between-measurement correlations. Nevertheless, for most of the sites, no group differences were observed in the strength of association between the MSEL and CDI. This suggests that the associations observed between the MSEL and CDI were not moderated by site-related differences, such as language spoken, culture, or overall differences in the demographics of the participants collected at each site. Overall, the similarities between infant groups and between sites in the extent to which MSEL and CDI scores correlate suggests that the cross-measure association is robust but moderate.

## Strengths and Limitations of the Study

A strength of this study were its sample size and the assessment of infant data from multiple perspectives and research sites. This allowed us to assess the generalizability of the results across multiple clinical and non-clinical infant groups, but also from different countries and languages.

However, although data was taken from multiple sites, and this provided a more comprehensive overview of our research question, all countries from which data was taken were European, containing samples of infants from societies that are western, educated, industrialised, rich, and democratic (WEIRD societies; Henrich et al., 2010). As such, the current findings may be less comparable to data from infants who grow up in non-WEIRD societies. Current initiatives to collect more data from non-WEIRD societies will help us to bridge these gaps and to gain a more comprehensive view of child development in the future (Broesch et al., 2016; Katus et al., 2024).

A limitation of the analyses was that the socio-economic status of the children (see Table 2) were not completely equal in the elevated compared to the typical likelihood groups, and in the autistic compared to non-autistic groups. We also evaluated only one age group, namely 14-month-olds. Therefore, we cannot investigate whether the observed concurrent validity changes across infancy. Another direction for future research will be to consider how the concurrent validity between the MSEL and CDI changes over the course of multiple time points.

An additional limitation was that the sample sizes differed across the countries. Moreover, although we used carefully constructed translations of the MSEL in each native language, these were not normed with the native populations. It could be that some milestones in communication are reached in some European languages earlier or later than in the American-English language (Hamilton et al., 2000; though see also Braginsky et al., 2019). As a result, these limitations might obscure language-related differences in the concurrent validity between the MSEL and the CDI.

Finally, it should be noted as a limitation that the MSEL testers may have known the likelihood classification of the child, as at some sites children and their families participated alongside the testers in a battery of tests for a full day. Knowing the classification of the child could have biased the testers in their behavioural assessments. However, the standardised nature in which the testing was carried out across the sites, along with the training that the researchers received in carrying out the assessment, served to minimise this effect (Jones et al., 2014).

## Conclusions and Practical Implications

We observed that during infancy, the concurrent validity between the MSEL and CDI was predominantly moderate in all groups, both likelihood (typical and elevated likelihood) and diagnostic (non-autistic and autistic). Based on these findings, care should be taken in generalising across the MSEL and CDI results of younger children. The measures may assess related but not identical constructs, and the way in which these constructs relate to each other could change over the course of development. Future psychometric studies should investigate the construct validity of these assessments in infants younger than 14 months, since they are often used to measure language abilities from 10 months onwards. Future studies could also examine latent factors to identify which assessment, or which items from multiple assessments, provide the best estimate of a child's true language abilities. It is further recommended that researchers consider the conceptual and methodological differences that exist between the measures when deciding which one to utilise in a research project, particularly if they plan to test younger children. Studies interested in both vocabulary size per se and linguistic capacities more broadly may wish to continue collecting both the MSEL and the CDI. This will provide a more comprehensive and accurate view of the child’s expressive and receptive skills than the use of solely one assessment or the other, by providing information about the child’s early language development from multiple raters and across multiple settings.

## Supplementary Information

Below is the link to the electronic supplementary material.

Supplementary file1 (DOCX 40 kb)

## Footnotes

1 In this study, it was necessary to know the genetic background of the children to classify them, meaning that all childrens’ caregivers were their biological parents. Therefore, the CDI is hereon referred to in this paper as being completed by the parents.

2 An exception was in the samples taken from BLINDED, who classified infants as elevated likelihood if any first degree relative (a parent or a sibling) had an autism diagnosis.

## References

[CR1] Akshoomoff, N. (2006). Use of the mullen scales of early learning for the assessment of young children with autism spectrum disorders. *Child Neuropsychology: A Journal on Normal and Abnormal Development in Childhood and Adolescence,**12*(4–5), 269–277. 10.1080/09297040500473714 16911972 10.1080/09297040500473714 PMC1550495

[CR2] Alcock, K., Meints, K., & Rowland, C. Brelsford, V., Christopher, A., & Just, J.. (2020). The UK communicative development inventories: Words and gestures. In: K. Alcock, K. Meints, C. Rowland (Eds). J&R Press. Retrieved from https://www.jr-press.co.uk/uk-communicative-development-inventories.html

[CR3] Aldridge, V. K., Dovey, T. M., & Wade, A. (2017). Assessing test-retest reliability of psychological measures. *European Psychologist,**22*(4), 207–218. 10.1027/1016-9040/a000298

[CR4] Bal, V. H., Fok, M., Lord, C., Smith, I. M., Mirenda, P., Szatmari, P., Vaillancourt, T., Volden, J., Waddell, C., Zwaigenbaum, L., Bennett, T., Duku, E., Elsabbagh, M., Georgiades, S., Ungar, W. J., & Zaidman-Zait, A. (2020). Predictors of longer-term development of expressive language in two independent longitudinal cohorts of language-delayed preschoolers with autism spectrum disorder. *Journal of Child Psychology and Psychiatry,**61*(7), 826–835. 10.1111/jcpp.13117 31429087 10.1111/jcpp.13117 PMC7028445

[CR5] Baranek, G. T. (1999). Autism during infancy: A retrospective video analysis of sensory-motor and social behaviors at 9–12 months of age. *Journal of Autism and Developmental Disorders,**29*(3), 213–224. 10.1023/A:1023080005650 10425584 10.1023/a:1023080005650

[CR6] Bates, E., Marchman, V., Thal, D., Fenson, L., Dale, P., Reznick, J. S., Reilly, J., & Hartung, J. (1994). Developmental and stylistic variation in the composition of early vocabulary. *Journal of Child Language,**21*(1), 85–123. 10.1017/S0305000900008680 8006096 10.1017/s0305000900008680

[CR7] Bayley, N. (2006). *Bayley scales of infant and toddler development, *3rd Edition. Retrieved from https://psycnet.apa.org/Landing?doi=10.1037%2Ft14978-000

[CR8] Belteki, Z., Lumbreras, R., Fico, K., Haman, E., & Junge, C. M. M. (2022). The Vocabulary of Infants with an elevated likelihood and diagnosis of autism spectrum disorder: A systematic review and meta-analysis of infant language studies using the CDI and MSEL. *International Journal of Environmental Research and Public Health,**19*(3), 1469. 10.3390/ijerph19031469 35162492 10.3390/ijerph19031469 PMC8834732

[CR9] Boucher, J. (2003). Language development in autism. *International Congress Series,**1254*, 247–253. 10.1016/S0531-5131(03)00976-2

[CR10] Bradley-Johnson, S. (1997). *Mullen scales of early learning*. 379–382.

[CR11] Braginsky, M., Yurovsky, D., Marchman, V. A., & Frank, M. C. (2019). Consistency and variability in children’s word learning across languages. *Open Mind,**3*, 52–67. 10.1162/opmi_a_00026 31517175 10.1162/opmi_a_00026 PMC6716390

[CR12] Bricker, D., Squires, J., Mounts, L., Potter, L., Nickel, R., Twombly, E., & Farrell, J. (1999). *Ages and stages questionnaire*. Brookes Publishing Company.

[CR13] Broesch, T., Rochat, P., Olah, K., Broesch, J., & Henrich, J. (2016). Similarities and differences in maternal responsiveness in three societies: Evidence from Fiji, Kenya, and the United States. *Child Development,**87*(3), 700–711. 10.1111/cdev.12501 27189398 10.1111/cdev.12501

[CR14] Canu, D., Van der Paelt, S., Canal-Bedia, R., Posada, M., Vanvuchelen, M., & Roeyers, H. (2021). Early non-social behavioural indicators of autism spectrum disorder (ASD) in siblings at elevated likelihood for ASD: A systematic review. *European Child & Adolescent Psychiatry,**30*(4), 497–538. 10.1007/s00787-020-01487-7 32088859 10.1007/s00787-020-01487-7 PMC8041710

[CR15] Charman, T. (2004). Matching preschool children with autism spectrum disorders and comparison children for language ability: Methodological challenges. *Journal of Autism and Developmental Disorders,**34*(1), 59–64. 10.1023/b:jadd.0000018075.77941.60 15098958 10.1023/b:jadd.0000018075.77941.60

[CR16] Charman, T., Drew, A., Baird, C., & Baird, G. (2003). Measuring early language development in preschool children with autism spectrum disorder using the MacArthur communicative development inventory (infant form). *Journal of Child Language,**30*, 213–236. 10.1017/S0305000902005482 12718299 10.1017/s0305000902005482

[CR17] Charman, T., Young, G. S., Brian, J., Carter, A., Carver, L. J., Chawarska, K., Curtin, S., Dobkins, K., Elsabbagh, M., Georgiades, S., Hertz-Picciotto, I., Hutman, T., Iverson, J. M., Jones, E. J., Landa, R., Macari, S., Messinger, D. S., Nelson, C. A., Ozonoff, S., & Zwaigenbaum, L. (2017). Non-ASD outcomes at 36 months in siblings at familial risk for autism spectrum disorder (ASD): A baby siblings research consortium (BSRC) study. *Autism Research: Official Journal of the International Society for Autism Research,**10*(1), 169–178. 10.1002/aur.1669 27417857 10.1002/aur.1669 PMC5993543

[CR18] Chiat, S., & Roy, P. (2007). The preschool repetition test: An evaluation of performance in typically developing and clinically referred children. *Journal of Speech, Language, and Hearing Research.*10.1044/1092-4388(2007/030) 10.1044/1092-4388(2007/030) 17463239

[CR19] Cohen, J. (1988). *Statistical power analysis for the behavioral sciences* (2nd ed.). Routledge. 10.4324/9780203771587

[CR20] Colombo, J. (2001). The development of visual attention in infancy. *Annual Review of Psychology,**52*(1), 337–367. 10.1146/annurev.psych.52.1.337 11148309 10.1146/annurev.psych.52.1.337

[CR21] Deanda, S., Arias-Trejo, N., Poulin-Dubois, D., Zesiger, P., & Friend, M. (2016). Minimal second language exposure, SES, and early word comprehension: New evidence from a direct assessment. *Bilingualism: Language and Cognition,**19*(1), 162–180. 10.1017/S1366728914000820 26957947 10.1017/S1366728914000820 PMC4779649

[CR22] Del Rosario, C., Nixon, E., Quigley, J., Whitehouse, A. J. O., & Maybery, M. T. (2023). Parent-child interaction and developmental outcomes in children with typical and elevated likelihood of autism. *Infant Behavior and Development,**71*, 101830. 10.1016/j.infbeh.2023.101830 36848788 10.1016/j.infbeh.2023.101830

[CR23] DesChamps, T. D., Ibañez, L. V., Edmunds, S. R., Dick, C. C., & Stone, W. L. (2020). Parenting stress in caregivers of young children with ASD concerns prior to a formal diagnosis. *Autism Research,**13*(1), 82–92. 10.1002/aur.2213 31593362 10.1002/aur.2213

[CR24] Edwards, S., Letts, C., & Sinka, I. (2011). New reynell developmental language scales. 10.1080/136828299247487 15587011

[CR25] Eriksson, M., & Berglund, E. (1999). Swedish early communicative development inventories: Words and gestures. *First Language,**19*(55), 55–90. 10.1177/014272379901905503

[CR26] Farmer, C., Golden, C., & Thurm, A. (2016). Concurrent validity of the differential ability scales, second edition with the Mullen scales of early learning in young children with and without neurodevelopmental disorders. *Child Neuropsychology,**22*(5), 556–569. 10.1080/09297049.2015.1020775 25833070 10.1080/09297049.2015.1020775 PMC5588686

[CR27] Feldman, H. M., Dollaghan, C. A., Campbell, T. F., Kurs-Lasky, M., Janosky, J. E., & Paradise, J. L. (2000). Measurement properties of the macarthur communicative development inventories at ages one and two years. *Child Development,**71*(2), 310–322. 10.1111/1467-8624.00146 10834466 10.1111/1467-8624.00146

[CR28] Fenson, L., Bates, E., Dale, P. S., Marchman, V. A., Reznick, J. S., & Thal, D. J. (2007). *MacArthur-bates communicative development inventories*. Brookes Publishing Company.

[CR29] Fenson, L., Dale, P. S., Reznick, J. S., Bates, E., Thal, D. J., Pethick, S. J., Tomasello, M., Mervis, C. B., & Stiles, J. (1994). Variability in early communicative development. *Monographs of the Society for Research in Child Development*. 10.2307/1166093 7845413

[CR30] Frank, M. C., Braginsky, M., Yurovsky, D., & Marchman, V. A. (2017). Wordbank: An open repository for developmental vocabulary data. *Journal of Child Language,**44*(3), 677–694. 10.1017/S0305000916000209 27189114 10.1017/S0305000916000209

[CR62] Frank, M. C., Braginsky, M., Yurovsky, D., & Marchman, V. A. (2021). *Variability and consistency in early language learning: The wordbank project*. MIT Press.

[CR31] Garrido, D., Petrova, D., Watson, L. R., Garcia-Retamero, R., & Carballo, G. (2017). Language and motor skills in siblings of children with autism spectrum disorder: A meta-analytic review. *Autism Research,**10*(11), 1737–1750. 10.1002/aur.1829 28685955 10.1002/aur.1829

[CR32] Hamilton, A., Plunkett, K., & Schafer, G. (2000). Infant vocabulary development assessed with a British communicative development inventory. *Journal of Child Language,**27*(3), 689–705. 10.1017/S0305000900004414 11089344 10.1017/s0305000900004414

[CR33] Hart, B., & Risley, T. R. (1995). *Meaningful differences in the everyday experience of young American children*. Paul H Brookes Publishing.

[CR34] Henrich, J., Heine, S. J., & Norenzayan, A. (2010). Most people are not WEIRD. *Nature,**466*(7302), 7302. 10.1038/466029a 10.1038/466029a 20595995

[CR67] Houston-Price, C., Mather, E., & Sakkalou, E. (2007). Discrepancy between parental reports of infants’ receptive vocabulary and infants’ behaviour in a preferential looking task. *Journal of Child Language,**34*(4), 701–724. 18062356 10.1017/s0305000907008124

[CR64] IBM Corp. (2023). *IBM SPSS Statistics for Windows (Version 29.0.0.0) [Computer software]*. IBM Corp. https://www.ibm.com/products/spss-statistics

[CR35] Iverson, J. M., Northrup, J. B., Leezenbaum, N. B., Parladé, M. V., Koterba, E. A., & West, K. L. (2018). Early gesture and vocabulary development in infant siblings of children with autism spectrum disorder. *Journal of Autism and Developmental Disorders,**48*(1), 55–71. 10.1007/s10803-017-3297-8 28900778 10.1007/s10803-017-3297-8 PMC5831718

[CR36] Jones, E., Gliga, T., Bedford, R., Charman, T., & Johnson, M. H. (2014). Developmental pathways to autism: A review of prospective studies of infants at risk. *Neuroscience & Biobehavioral Reviews,**39*, 1–33. 10.1016/j.neubiorev.2013.12.001 24361967 10.1016/j.neubiorev.2013.12.001 PMC3969297

[CR60] Jones, E. J., Mason, L., Ali, J. B., Van Den Boomen, C., Braukmann, R., Cauvet, E., & Johnson, M. H. (2019). Eurosibs: Towards robust measurement of infant neurocognitive predictors of autism across Europe. *Infant Behavior and Development,**57*, 101316. 31128517 10.1016/j.infbeh.2019.03.007 PMC6891238

[CR37] Katus, L., Crespo-Llado, M. M., Milosavljevic, B., Saidykhan, M., Njie, O., Fadera, T., McCann, S., Acolatse, L., Perapoch Amadó, M., Rozhko, M., Moore, S. E., Elwell, C. E., & Lloyd-Fox, S. (2024). It takes a village: Caregiver diversity and language contingency in the UK and rural Gambia. *Infant Behavior and Development,**74*, 101913. 10.1016/j.infbeh.2023.101913 38056188 10.1016/j.infbeh.2023.101913

[CR38] Keating, C. T., Hickman, L., Leung, J., Monk, R., Montgomery, A., Heath, H., & Sowden, S. (2023). Autism-related language preferences of English-speaking individuals across the globe: A mixed methods investigation. *Autism Research,**16*(2), 406–428. 36474364 10.1002/aur.2864 PMC10946540

[CR61] Kuhl, P. K. (2004). Early language acquisition: Cracking the speech code. *Nature reviews neuroscience,**5*(11), 831–843. 15496861 10.1038/nrn1533

[CR39] Kwok, E. Y. L., Brown, H. M., Smyth, R. E., & Oram Cardy, J. (2015). Meta-analysis of receptive and expressive language skills in autism spectrum disorder. *Research in Autism Spectrum Disorders,**9*, 202–222. 10.1016/j.rasd.2014.10.008

[CR40] Landa, R. J., Holman, K. C., & Garrett-Mayer, E. (2007). Social and communication development in toddlers with early and later diagnosis of autism spectrum disorders. *Archives of General Psychiatry,**64*(7), 853–864. 10.1001/archpsyc.64.7.853 17606819 10.1001/archpsyc.64.7.853

[CR41] Lord, C., & Luyster, R. (2006). Early diagnosis of children with autism spectrum disorders. *Clinical Neuroscience Research,**6*(3), 189–194. 10.1016/j.cnr.2006.06.005

[CR42] Lord, C., Risi, S., Lambrecht, L., Cook, E. H., Leventhal, B. L., DiLavore, P. C., Pickles, A., & Rutter, M. (2000). The autism diagnostic observation schedule—generic: A standard measure of social and communication deficits associated with the spectrum of autism. *Journal of Autism and Developmental Disorders,**30*(3), 205–223. 10.1023/A:1005592401947 11055457

[CR43] Marrus, N., Hall, L. P., Paterson, S. J., Elison, J. T., Wolff, J. J., Swanson, M. R., Parish-Morris, J., Eggebrecht, A. T., Pruett, J. R., Hazlett, H. C., Zwaigenbaum, L., Dager, S., Estes, A. M., Schultz, R. T., Botteron, K. N., Piven, J., Constantino, J. N., & Network, I. (2018). Language delay aggregates in toddler siblings of children with autism spectrum disorder. *Journal of Neurodevelopmental Disorders,**10*(1), 1. 30348077 10.1186/s11689-018-9247-8 PMC6198516

[CR65] McDade, H. L., Simpson, M. A., & Lamb, D. E. (1982). The use of elicited imitation as a measure of expressive grammar: A question of validity. *Journal of Speech and Hearing disorders,**47*(1), 19–24. 7176570 10.1044/jshd.4701.19

[CR44] Mullen, E. M. (1995). *Mullen scales of early learning*. American Guidance Service.

[CR45] Nordahl-Hansen, A., Kaale, A., & Ulvund, S. E. (2014). Language assessment in children with autism spectrum disorder: Concurrent validity between report-based assessments and direct tests. *Research in Autism Spectrum Disorders,**8*(9), 1100–1106. 10.1016/j.rasd.2014.05.017

[CR46] Osterling, J. A., Dawson, G., & Munson, J. A. (2002). Early recognition of 1-year-old infants with autism spectrum disorder versus mental retardation. *Development and Psychopathology,**14*(2), 239–251. 10.1017/S0954579402002031 12030690 10.1017/s0954579402002031

[CR47] Ozonoff, S., Young, G. S., Bradshaw, J., Charman, T., Chawarska, K., Iverson, J. M., Klaiman, C., Landa, R. J., McDonald, N., Messinger, D., Schmidt, R. J., Wilkinson, C. L., & Zwaigenbaum, L. (2024). Familial recurrence of Autism: Updates from the baby siblings research consortium. *Pediatrics,**154*(2), e2023065297. 10.1542/peds.2023-065297 39011552 10.1542/peds.2023-065297 PMC11291960

[CR48] Ozonoff, S., Young, G. S., Carter, A., Messinger, D., Yirmiya, N., Zwaigenbaum, L., Bryson, S., Carver, L. J., Constantino, J. N., Dobkins, K., Hutman, T., Iverson, J. M., Landa, R., Rogers, S. J., Sigman, M., & Stone, W. L. (2011). Recurrence risk for autism spectrum disorders: A baby siblings research consortium study. *Pediatrics,**128*(3), e488–e495. 10.1542/peds.2010-2825 21844053 10.1542/peds.2010-2825 PMC3164092

[CR49] Rescorla, L., Ratner, N. B., Jusczyk, P., & Jusczyk, A. M. (2005). Concurrent validity of the language development survey: Associations with the macarthur—bates communicative development inventories. *American Journal of Speech-Language Pathology,**14*(2), 156–163. 10.1044/1058-0360(2005/016) 15989390 10.1044/1058-0360(2005/016)

[CR50] Riley, E., Paynter, J., & Gilmore, L. (2019). Comparing the mullen scales of early learning and the preschool language scale—fifth edition for young children with autism spectrum disorder. *Advances in Neurodevelopmental Disorders,**3*(1), 29–37. 10.1007/s41252-018-0084-2

[CR51] Rose, S. A., Blank, M., & Spalter, I. (1975). Situational specificity of behavior in young children. *Child Development,**46*(2), 464–469. 10.2307/1128143

[CR63] R Studio Team. (2023). RStudio: Integrated Development Environment for R (Version 1.4.1103) [Computer software]. RStudio, PBC. https://posit.co/downloads/

[CR52] Rutter, M., Le Couteur, A., & Lord, C. (2003). *Autism diagnostic interview-revised*. Western Psychological Services.

[CR53] Schönbrodt, F. D., & Perugini, M. (2013). At what sample size do correlations stabilize? *Journal of Research in Personality,**47*(5), 609–612. 10.1016/j.jrp.2013.05.009

[CR54] Smoczyńska, M., Krajewski, G., Łuniewska, M., Haman, E., Bulkowski, K., & Kochańska, M. (2015). *Inwentarze rozwoju mowy i komunikacji (IRMIK) słowa i gesty, słowa i zdania: Podręcznik*. Instytut Badań Edukacyjnych.

[CR55] Sparrow, S. S., Balla, D. A., & Cicchetti, D. V. (2005). *Vineland II: Vineland adaptive behavior scales*. American Guidance Service.

[CR56] Swanson, M. R., Donovan, K., Paterson, S., Wolff, J. J., Parish-Morris, J., Meera, S. S., Watson, L. R., Estes, A. M., Marrus, N., Elison, J. T., Shen, M. D., McNeilly, H. B., MacIntyre, L., Zwaigenbaum, L., St John, T., Botteron, K., Dager, S., Piven, J., & Network, I. (2019). Early language exposure supports later language skills in infants with and without autism. *Autism Research: Official Journal of the International Society for Autism Research,**12*(12), 12. 10.1002/aur.2163 PMC6954821 31254329

[CR57] Thal, D., DesJardin, J. L., & Eisenberg, L. S. (2007). Validity of the macarthur–bates communicative development inventories for measuring language abilities in children with cochlear implants. *American Journal of Speech-Language Pathology,**16*(1), 54–64. 10.1044/1058-0360(2007/007) 17329675 10.1044/1058-0360(2007/007)

[CR58] Tomasello, M., & Mervis, C. B. (1994). The instrument is great, but measuring comprehension is still a problem. *Monographs of the Society for Research in Child Development,**59*(5), 174–179. 10.1111/j.1540-5834.1994.tb00186.x

[CR66] Vinther, T. (2002). Elicited imitation: A brief overview. *International Journal of Applied Linguistics,**12*(1), 54–73.

[CR59] Zink, I., & Lejaegere, M. (2002). *N-CDIs: Lijsten voor communicatieve ontwikkeling [NCDIs: Lists for communicative development]*. Acco.

