# Supplementary material for: A Concurrent Validity Study of the Mullen Scales of Early Learning (MSEL) and the MacArthur-Bates Communicative Developmental Inventory (CDI) in Infants with an Elevated Likelihood or Diagnosis of Autism
Source: J Autism Dev Disord. 2025 Jan 17;56(6):2323–38. doi: 10.1007/s10803-024-06652-4 (PMC13222192; doi:10.1007/s10803-024-06652-4)
Supplement: Supplementary file 1 — Supplementary file1 (DOCX 40 kb) [file 10803_2024_6652_MOESM1_ESM.docx]

**Supplementary material A**

Below are the results when the MacArthur-Bates Communicative Developmental Inventory (CDI) was converted to proportions. This was done to remove differences in scale across different language versions of the questionnaire.

**Group differences in language scores**

***Likelihood group comparisons***

The scores of typical likelihood infants on the CDI were not significantly higher than the scores of elevated likelihood infants, neither in receptive language (*z* = -1.63, *p* = .103, η^2^ **=** .066), nor in expressive language (z = -.14, *p* = .89, η^2^ **=** .0056).

***Diagnostic group comparisons***

The scores of non-autistic infants on the CDI were significantly higher than the scores of autistic infants, in both their receptive language (*z* = -3.28, *p* < .001, η^2^ **=** .14), and also in their expressive language (*z* = -3.73, *p* < .001, η^2^ = .15).

**Correlations between the CDI and MSEL assessments**

***Likelihood group comparisons***

For expressive scores, there was a moderate positive correlation between the CDI and MSEL for the typical likelihood group, *rs*(169) = .44, *p* < .001. There was also a moderate but higher positive correlation between the CDI and MSEL for the elevated likelihood group, *rs*(421) = .57, *p* < .001. The correlation for the elevated likelihood group was significantly higher than that for the typical likelihood group, *z* = -1.92, *p* = .027.

For receptive scores, there was a moderate positive correlation between the CDI and MSEL for the typical likelihood group, r*s*(169) = .38, *p* < .001. There was also a moderate correlation between the CDI and MSEL for the elevated likelihood group, r*s*(422) = .45, *p* < .001. The correlations were not significantly different between the typical likelihood and elevated likelihood groups, *z* = -.93., *p* = .18.

***Diagnostic group comparisons***

For expressive scores, there was a moderate positive correlation between the CDI and MSEL for the non-autistic group, r*s*(432) = .53, *p* < .001*.* There was also a moderate positive correlation between the CDI and MSEL for the autistic group, r*s*(82) = .44, *p* < .001. The correlation for the non-autistic group was not significantly different between the non-autistic and autistic groups, *z* = .97., *p* = .17.

For receptive scores, there was a moderate positive correlation between the CDI and MSEL for the non-autistic group, *rs*(433) = .43, *p* < .001. There was also a moderate correlation between the CDI and MSEL for the autistic group, *rs*(82) = .35, *p* < .001. The correlations were not significantly different between the non-autistic and autistic groups, *z* = .78, *p* = .22.

**Supplementary material B**

Below are the results when the MacArthur-Bates Communicative Developmental Inventory (CDI) was converted to proportions and both the Mullen Scales of Early Learning (MSEL) and CDI outliers were removed. Outliers were defined as any participant score that was below the 1^st^ percentile, or above the 99^th^ percentile. The number of outliers removed for the MSEL and CDI in the likelihood and the diagnostic groups is provided in Table 5.

**Group differences in language scores (converted to proportions, outliers removed).**

***Likelihood group comparisons***

The scores of typical likelihood infants on the MSEL were significantly higher than the scores of elevated likelihood infants, both in expressive language (*z* = -3.17, *p* = .002, η^2^ = .13), and in receptive language, (*z* = -2.80, *p* = .005, η^2^ = .11).

The scores of typical likelihood infants on the CDI were not significantly higher than the scores of elevated likelihood infants, neither in in expressive language (*z* = -.099, *p* = .92, η^2^ **=** .0040), nor in receptive language (*z* = -1.66, *p* = .097, η^2^ **=** .067).

***Diagnostic group comparisons***

The scores of non-autistic infants on the MSEL were significantly higher than the scores of autistic infants, both in expressive language (*z* = -5.44, *p* < .001, η^2^ = .23), and in receptive language (*z* = -4.24, *p* < .001 , η^2^ **=** .18).

The scores of non-autistic infants were significantly higher than the scores of autistic infants on the CDI, both in expressive language (*z* = -3.44, *p* < .001, η^2^ = .15), and in receptive language, (*z* = -3.33, *p* < .001 , η^2^ = .15).

**Correlations between MSEL and CDI assessments (when the CDI is converted to proportions and the outliers from both the MSEL and CDI are removed)**

***Likelihood group comparisons***

For expressive scores, there was a moderate positive correlation between the MSEL and CDI for the typical likelihood group, *rs*(164) = .43, *p* < .001. For the elevated likelihood group, there was also a moderate positive correlation between the MSEL and CDI, *rs*(411) = .55, *p* < .001. The correlation was significantly higher for the elevated likelihood group than in the typical likelihood group, *z* = -1.71, *p* = .044.

For receptive scores, there was a moderate positive correlation between the MSEL and CDI for the typical likelihood group, r*s*(163) = .41, *p* < .001. There was also a moderate correlation between the MSEL and CDI for the elevated likelihood group, r*s*(411) = .44, *p* < .001. The correlations were not significantly different between the typical likelihood and elevated likelihood groups, *z* = -.39., *p* = .35.

***Diagnostic group comparisons***

For expressive scores, there was a moderate positive correlation between the MSEL and CDI for the non-autistic group, *rs*(419) = .51 *p* < .001. There was also a moderate but higher positive correlation between the MSEL and CDI for the autistic group, *rs*(79) = .41, *p* < .001. The correlations were not significantly different between the non-autistic and autistic groups, *z* = 1.02, *p* = .15.

For receptive scores, there was a moderate positive correlation between the MSEL and CDI for the non-autistic group, r*s*(419) = .43, *p* < .001. There was also a moderate correlation between the MSEL and CDI for the autistic group, r*s*(80) = .32, *p* < .001. The correlations were not significantly different between the non-autistic and autistic infant groups, *z* = 1.04., *p* = .15.

**Table 5.**

*The number of participants removed in the likelihood and diagnostic groups for the MSEL and CDI.*

|  | Likelihood groups | | Diagnostic groups | |
| --- | --- | --- | --- | --- |
|  | Typical likelihood | Elevated likelihood | Non-autistic | Autistic |
| MSEL | 8 (4 receptive) | 14 (8 receptive) | 19 (10 receptive) | 3 (1 receptive) |
| CDI | 4 (2 receptive) | 9 (5 receptive) | 10 (5 receptive) | 2 (1 receptive) |

**Supplementary material C: Gender**

We ran additional analyses examining the gender differences in the concurrent validity between the Mullen Scales of Early Learning (MSEL) and the MacArthur-Bates Communicative Developmental Inventory (CDI). This information is summarised in Table 6 and Table 7 on the next page.

The concurrent validity between the MSEL and the CDI did not differ significantly for the majority of pre-diagnostic (EL versus TL) and post-diagnostic comparisons (autistic versus non-autistic). However, the MSEL and CDI expressive scores of typical likelihood girls had a significantly lower concurrent validity than the MSEL and CDI expressive scores of elevated likelihood girls.

**Table 6**

*The concurrent validity between the MSEL and CDI for expressive scores, split by gender.*

| **Expressive vocabulary** | Likelihood groups | | | | Diagnostic groups | | | |  |
| --- | --- | --- | --- | --- | --- | --- | --- | --- | --- |
| Gender | | Typical likelihood | Elevated likelihood | **Group difference** | | Non-autistic | Autistic | **Group difference** | |
| Boys | | *rs* (87) = .46** | *rs* (227) = .55** | *z* = -0.95 | | *rs* (214) = .53** | *rs* (57) = .38** | *z* = 1.29 | |
| Girls | | *rs* (83) = .38** | *rs* (201) = .61** | *z* = -2.31* | | *rs* (219) = .54** | *rs* (27) = .59** | *z* = -0.33 | |
| **Group difference** | | z = 0.67 | z = -0.83 |  | | z = -0.12 | z = -1.14 |  | |

*NB:*  ** marks significance at p < .05) and ** at p <.01.*

**Table 7**

*The concurrent validity between the MSEL and CDI for receptive scores, split by gender.*

| **Receptive vocabulary** | Likelihood groups | | | | Diagnostic groups | | | |  |
| --- | --- | --- | --- | --- | --- | --- | --- | --- | --- |
| Gender | | Typical likelihood | Elevated likelihood | **Group difference** | | Non-autistic | Autistic | **Group difference** | |
| Boys | | *rs* (87) = .31** | *rs* (221) = .44** | *z* = -1.19 | | *rs* (216) = .39** | *rs* (56) = .23 | *z* = 1.20 | |
| Girls | | *rs* (83) = .46** | *rs* (202) = .47** | *z* = -0.13 | | *rs* (223) = .50** | *rs* (27) = .44* | *z* = 0.34 | |
| **Group difference** | | z = -1.13 | z = -0.43 |  | | z = -1.33 | z = -0.98 |  | |

*NB:*  ** marks significance at p < .05) and ** at p <.01.*

**Supplementary material D: Language**

We ran additional analyses examining the language differences in the concurrent validity between the Mullen Scales of Early Learning (MSEL) and the MacArthur-Bates Communicative Developmental Inventory (CDI). This information is summarised in Table 8 and Table 9 on the next page.

The concurrent validity between the MSEL and the CDI did not differ significantly for the majority of pre-diagnostic (EL versus TL) and post-diagnostic comparisons (autistic versus non-autistic). An exception was that the elevated likelihood groups’ expressive scores had a higher concurrent validity than the expressive scores of the typical likelihood group. This was only observed in the English sample, and not the samples who spoke other languages. For the most part, the English sample and the other languages sample were comparable in the concurrent validity scores of most infant group (elevated, typical, non-autistic, autistic). However, two exceptions were observed when looking at receptive vocabulary (Table 9). The first was that the elevated likelihood group taken from the English sample had a lower concurrent validity than the elevated likelihood group taken from the other languages sample. The second was that the non-autistic group taken from the English sample had a lower concurrent validity than the non-autistic group taken from the other languages sample.

**Table 8**

*The concurrent validity between the MSEL and CDI for expressive scores, split by language.*

| **Expressive vocabulary** | Likelihood groups | | | | Diagnostic groups | | | |  |
| --- | --- | --- | --- | --- | --- | --- | --- | --- | --- |
| Language | | Typical likelihood | Elevated likelihood | **Group difference** | | Non-autistic | Autistic | **Group difference** | |
| English | | *rs* (96) = .40** | *rs* (250) = .61** | *z* = -2.42** | | *rs* (236) = .53** | *rs* (44) = .49** | *z* = 0.32 | |
| Other languages | | *rs* (74) = .52** | *rs* (172) = .50** | *z* = 0.191 | | *rs* (197) = .56** | *rs* (39) = .33** | *z* = 1.62 | |
| **Group difference** | | z = -1.02 | z = 1.60 |  | | z = -0.48 | z = 0.85 |  | |

*NB:*  ** marks significance at p < .05) and ** at p <.01.*

**Table 9**

*The concurrent validity between the MSEL and CDI for receptive scores, split by language.*

| **Receptive vocabulary** | Likelihood groups | | | | Diagnostic groups | | | |  |
| --- | --- | --- | --- | --- | --- | --- | --- | --- | --- |
| Language | | Typical likelihood | Elevated likelihood | **Group difference** | | Non-autistic | Autistic | **Group difference** | |
| English | | *rs* (96) = .37** | *rs* (251) = .40** | *z* = -0.25 | | *rs* (237) = .37** | *rs* (44) = .25 | *z* = 0.78 | |
| Other languages | | *rs* (74) = .46** | *rs* (172) = .55** | *z* = -0.875 | | *rs* (197) = .55** | *rs* (39) = .47** | *z* = 0.62 | |
| **Group difference** | | z = -0.68 | z = -2.01* |  | | z = -2.38** | z = -1.10 |  | |

*NB:*  ** marks significance at p < .05) and ** at p <.01.*

**Supplementary material E: Socio-economic status (SES)**

We ran additional analyses examining the socio-economic status differences in the concurrent validity between the Mullen Scales of Early Learning (MSEL) and the MacArthur-Bates Communicative Developmental Inventory (CDI). This information is summarised in Table 10 and Table 11 on the next page.

The concurrent validity between the MSEL and the CDI did not differ significantly for the majority of pre-diagnostic (EL versus TL) and post-diagnostic comparisons (autistic versus non-autistic). An exception was that the elevated likelihood groups’ expressive scores had a higher concurrent validity than the expressive scores of the typical likelihood group. This was only observed in the tertiary sample, and not the secondary sample. The secondary and tertiary samples were comparable in the concurrent validity scores of most infant groups (elevated, typical, non-autistic, autistic). However, two exceptions were observed when looking at receptive vocabulary (Table 11). The first was that the elevated likelihood group taken from the Tertiary sample had a higher concurrent validity than the elevated likelihood group taken from the Secondary sample. The second was that the non-autistic group taken from the Tertiary sample had a higher concurrent validity than the non-autistic group taken from the Secondary sample.

**Table 10**

*The concurrent validity between the MSEL and CDI for expressive scores, split by SES.*

| **Expressive vocabulary** | Likelihood groups | | | | Diagnostic groups | | | |  |
| --- | --- | --- | --- | --- | --- | --- | --- | --- | --- |
| SES | | Typical likelihood | Elevated likelihood | **Group difference** | | Non-autistic | Autistic | **Group difference** | |
| Secondary | | *rs* (25) = .52** | *rs* (126) = .54** | *z* = -0.098 | | *rs* (93) = .54** | *rs* (32) = .51** | *z* = 0.19 | |
| Tertiary | | *rs* (123) = .49** | *rs* (208) = .60** | *z* = -1.36 | | *rs* (265) = .54** | *rs* (24) = .55** | *z* = -0.11 | |
| **Group difference** | | z = 0.17 | z = -0.83 |  | | z = 0.002 | z = -0.24 |  | |

*NB:*  ** marks significance at p < .05) and ** at p <.01.*

**Table 11**

*The concurrent validity between the MSEL and CDI for receptive scores, split by SES.*

| **Receptive vocabulary** | Likelihood groups | | | | Diagnostic groups | | | |  |
| --- | --- | --- | --- | --- | --- | --- | --- | --- | --- |
| SES | | Typical likelihood | Elevated likelihood | **Group difference** | | Non-autistic | Autistic | **Group difference** | |
| Secondary | | *rs* (25) = .29** | *rs* (126) = .20** | *z* = 0.45 | | *rs* (93) = .27* | *rs* (32) = .30 | *z* = -0.36 | |
| Tertiary | | *rs* (123) = .41** | *rs* (209) = .56** | *z* = -1.74* | | *rs* (266) = .49** | *rs* (24) = .23 | *z* = 1.32 | |
| **Group difference** | | z = -0.58 | z = -3.85** |  | | z = -2.47** | z = 0.26 |  | |

*NB:*  ** marks significance at p < .05) and ** at p <.01.*
